# Fractal dimension of the cortical gray matter outweighs other brain MRI features as a predictor of transition to dementia in patients with mild cognitive impairment and leukoaraiosis

**DOI:** 10.3389/fnhum.2023.1231513

**Published:** 2023-09-26

**Authors:** Chiara Marzi, Riccardo Scheda, Emilia Salvadori, Antonio Giorgio, Nicola De Stefano, Anna Poggesi, Domenico Inzitari, Leonardo Pantoni, Mario Mascalchi, Stefano Diciotti

**Affiliations:** ^1^Department of Statistics, Computer Science, Applications “Giuseppe Parenti, ” University of Florence, Florence, Italy; ^2^Department of Electrical, Electronic, and Information Engineering “Guglielmo Marconi, ” University of Bologna, Cesena, Italy; ^3^NEUROFARBA Department, Neuroscience Section, University of Florence, Florence, Italy; ^4^Department of Medicine, Surgery, and Neuroscience, University of Siena, Siena, Italy; ^5^Department of Biomedical and Clinical Sciences, University of Milan, Milan, Italy; ^6^Department of Experimental and Clinical Biomedical Sciences “Mario Serio, ” University of Florence, Florence, Italy; ^7^Division of Epidemiology and Clinical Governance, Institute for Study, Prevention and Network in Oncology (ISPRO), Florence, Italy; ^8^Alma Mater Research Institute for Human-Centered Artificial Intelligence, University of Bologna, Bologna, Italy

**Keywords:** dementia, fractal dimension, gray matter, leukoaraiosis, mild cognitive impairment, MRI, white matter

## Abstract

**Background:**

The relative contribution of changes in the cerebral white matter (WM) and cortical gray matter (GM) to the transition to dementia in patients with mild cognitive impairment (MCI) is not yet established. In this longitudinal study, we aimed to analyze MRI features that may predict the transition to dementia in patients with MCI and T_2_ hyperintensities in the cerebral WM, also known as leukoaraiosis.

**Methods:**

Sixty-four participants with MCI and moderate to severe leukoaraiosis underwent baseline MRI examinations and annual neuropsychological testing over a 2 year period. The diagnosis of dementia was based on established criteria. We evaluated demographic, neuropsychological, and several MRI features at baseline as predictors of the clinical transition. The MRI features included visually assessed MRI features, such as the number of lacunes, microbleeds, and dilated perivascular spaces, and quantitative MRI features, such as volumes of the cortical GM, hippocampus, T_2_ hyperintensities, and diffusion indices of the cerebral WM. Additionally, we examined advanced quantitative features such as the fractal dimension (FD) of cortical GM and WM, which represents an index of tissue structural complexity derived from 3D-T_1_ weighted images. To assess the prediction of transition to dementia, we employed an XGBoost-based machine learning system using SHapley Additive exPlanations (SHAP) values to provide explainability to the machine learning model.

**Results:**

After 2 years, 18 (28.1%) participants had transitioned from MCI to dementia. The area under the receiving operator characteristic curve was 0.69 (0.53, 0.85) [mean (90% confidence interval)]. The cortical GM-FD emerged as the top-ranking predictive feature of transition. Furthermore, aggregated quantitative neuroimaging features outperformed visually assessed MRI features in predicting conversion to dementia.

**Discussion:**

Our findings confirm the complementary roles of cortical GM and WM changes as underlying factors in the development of dementia in subjects with MCI and leukoaraiosis. FD appears to be a biomarker potentially more sensitive than other brain features.

## 1. Introduction

Mild cognitive impairment (MCI) is a condition characterized by a variable impairment of cognitive functions that does not interfere with activities of daily living (Gauthier et al., 2006). Over half of the subjects with MCI progress to dementia in the next 5 years (Gauthier et al., 2006). Since vascular and neurodegenerative diseases overlap in the older population and both may underlie MCI and dementia (Jellinger, 2013), distinguishing the respective contributors to the transition to dementia can be difficult. There is great interest in the identification of biomarkers as predictors of the transition to dementia in patients with MCI in longitudinal studies (Jokinen et al., 2012, 2015, 2020; Bilello et al., 2015; Wright and Flores, 2015; Ye et al., 2015; Williams et al., 2017, 2019; Zeestraten et al., 2017; Wu et al., 2019; Egle et al., 2022). Changes in the cerebral subcortical white matter (WM) appearing as areas of decreased density on computed tomography or hyperintensities on T_2_-weighted MR images, termed leukoaraiosis, are associated with changes in the diffusion of water protons in both T_2_-weighted hyperintense and normal-appearing WM (O'Sullivan, 2008). Such WM changes are a common finding in elderly subjects whose cognitive functions span from normal to MCI and dementia (Fazekas et al., 1987; Golomb et al., 1995; O'Sullivan, 2008; Inzitari et al., 2009). Leukoaraiosis, along with lacunes and microbleeds, is a marker of small vessel disease (SVD) (Jokinen et al., 2015, 2020; Lambert et al., 2016; Williams et al., 2017, 2019; Zeestraten et al., 2017), but, overall, it is a non-specific finding that is observed in elderly subjects with preserved cognition and patients with Alzheimer's disease (AD) (Fazekas et al., 1987; Golomb et al., 1995; Bracco et al., 2005; O'Sullivan, 2008; Bilello et al., 2015).

The Vascular MCI (VMCI) Tuscany study aimed to identify clinical, neuroimaging, and biological markers predictive of transition to dementia in patients with MCI and leukoaraiosis (Poggesi et al., 2012). In the VMCI Tuscany study, visually assessed MRI features of brain damage included the number of lacunes, microbleeds (Valenti et al., 2016), and dilated perivascular spaces (Mascalchi et al., 2014). Quantitative MRI assessment included volumes of the entire cortical gray matter (GM), hippocampus, and T_2_ hyperintense WM (Giorgio et al., 2019) and diffusion properties of the T_2_ hyperintense and normal-appearing WM (Mascalchi et al., 2014; Ciulli et al., 2016). We also considered advanced quantitative features such as the fractal dimension (FD) of the cortical GM and WM (Pantoni et al., 2019)—indices of tissue structural complexity extracted from 3D-T_1_ weighted images (Marzi et al., 2020).

Herein, our objective was to assess the predictive power for the transition to dementia of various factors, including demographic data, neuropsychological assessments, and both visually and quantitatively assessed MRI features over a 2-year period. This evaluation was conducted on a cohort of 64 patients with MCI and leukoaraiosis as part of the VMCI Tuscany study.

## 2. Materials and methods

### 2.1. Patients

The VMCI Tuscany study was approved by the local ethical committees of the three participating centers in the Tuscany region of Italy, namely Florence, Pisa, and Siena, which shared selection criteria and assessment protocols (Poggesi et al., 2012).

The inclusion criteria for the VMCI Tuscany study were as follows: (1) MCI as defined according to the criteria by Winblad et al. (2004), and (2) evidence of moderate to severe T_2_ hyperintensity in the cerebral WM, based on the modified version of the Fazekas scale (Pantoni et al., 2005). The VMCI Tuscany study recruited 138 subjects, all of whom provided written informed consent to participate in the study (Salvadori et al., 2018).

Each patient underwent a comprehensive neuropsychological evaluation developed for patients with SVD and MCI (Salvadori et al., 2016), including both global cognitive functioning tests (i.e., Montreal Cognitive Assessment (MoCA) (Nasreddine et al., 2005; Conti et al., 2015) and second-level tests covering different cognitive domains [i.e., Visual Search (VS) (Della Sala et al., 1992), Symbol-Digit Modalities Test (SDMT) (Nocentini et al., 2006), Trail-Making Test (TMT), Part A (Giovagnoli et al., 1996), Color Word Stroop Test (Stroop) (Caffarra et al., 2002), and an immediate copy of the Rey–Osterrieth Complex Figure (ROC-F)]. For the neuropsychological tests, we used the available normative data based on healthy Italian adult samples' national norms to calculate demographically adjusted scores using the regression equations extracted by normative studies (details in Pantoni et al., 2019). The definition of change from MCI to dementia, or major neurocognitive disorder, was conducted in accordance with the DSM-5 criteria (Salvadori et al., 2018). The results of the baseline clinical, neuropsychological (Salvadori et al., 2016, 2018), and MRI assessments have been reported in previous studies (Valenti et al., 2016; Giorgio et al., 2019).

For this study, 64 participants were selected from a single center, and they underwent baseline MRI and annual neuropsychological testing over a 2-year period (Table 1). After 2 years, 18 (28.1%) participants had converted from MCI to dementia. The results of the cross-sectional assessment in this sub-cohort using visually assessed and quantitative MRI features and their correlation with the neuropsychological evaluation were reported in a previous study (Pantoni et al., 2019).

**Table 1:** Descriptive statistics of demographic, neuropsychological, visually assessed MRI, and quantitative MRI features for patients with and without a 2-year transition to dementia.

	**Feature**	**Patients without transition to dementia (*N* = 46)**	**Patients with transition to dementia (*N* = 18)**
Demographic	Age	73.96 (6.67) [61.12, 89.03]	76.34 (6.693) [59.80, 84.09]
	Sex	22 female and 24 male patients	8 female and 10 male patients
	Education	8.17 (4.25) [3, 18]	7.44 (4.30) [2, 18]
Neuropsychological test	MoCA	21.23 (4.62) [11.95, 29.29]	18.93 (3.95) [13.10, 25.24]
	ROC-F immediate copy	23.68 (7.21) [5.59, 35.58]	21.27 (10.61) [4, 36]
	SDMT	39.18 (10.03) [22.02, 59.94]	31.18 (5.38) [24.67, 43.49]
	Stroop	33.44 (23.81) [−3.45, 114.57]	51.59 (36.02) [8.83, 155.09]
	TMT-A	61.47 (47.97) [3.77, 202.2]	64.47 (43.15) [8.42, 152.92]
	VS	32.84 (8.61) [14.3, 50.17]	29.08 (7.78) [15.41, 41.27]
Visually assessed MRI features	Lacunar infarcts	2.02 (0.80) [1, 3]	2.28 (0.83) [1, 3]
	Cerebral microbleeds	0.91 (2.57) [0, 15]	2.24 (5.77) [0, 18]
	EPVS basal ganglia	1.67 (0.82) [0, 4]	1.83 (0.62) [1, 3]
	EPVS centrum semiovale	1.89 (0.77) [1, 3]	1.44 (0.70) [1, 3]
Quantitative MRI features	WM lesion load	0.07 (0.04) [0.01, 0.20]	0.09 (0.05) [0.02, 0.20]
	WM volume	0.15 (0.01) [0.12, 0.17]	0.14 (0.01) [0.13, 0.16]
	GM volume	0.12 (0.01) [0.10, 0.14]	0.11 (0.01) [0.10, 0.12]
	Hippocampal volume	0.0023 (0.0005) [0.0010, 0.0031]	0.0020 (0.0002) [0.0020, 0.0024]
	WM FD	2.45 (0.04) [2.35, 2.51]	2.43 (0.04) [2.36, 2.49]
	GM FD	2.34 (0.02) [2.30, 2.38]	2.33 (0.02) [2.27, 2.36]
	Median FA	0.37 (0.02) [0.33, 0.41]	0.36 (0.02) [0.32, 0.40]
	Median MD	0.82 (0.05) [0.7, 0.9]	0.82 (0.04) [0.8, 0.9]

The statistics are reported as the mean values (standard deviation) [minimum and maximum values] in the respective groups. The volume and the FD of a specific brain structure are defined as the average value between the left and right hemispheres. Volumes were subsequently normalized to eTIV. EPVS, enlarged perivascular spaces; FA, fractal anisotropy; FD, fractal dimension; GM, gray matter; MD, mean diffusivity; MoCA, adjusted Montreal Cognitive Assessment score; ROC-F, adjusted Rey-Osterrieth Complex Figure immediate copy score; SDMT, adjusted symbol-digit modality test score; TMT-A, adjusted trail-making test-A score; VS, adjusted visual search score; WM, white matter.

### 2.2. MRI acquisition protocol

All examinations were performed on a 1.5 T system (Intera, Philips Medical System, Best, The Netherlands) with 33 mT/m maximum gradient strength and a 6-channel head coil with SENSE technology. After scouts, we obtained sagittal 3D T_1_-weighted turbo gradient echo [repetition time (TR) = 8.1 ms, echo time (TE) = 3.7 ms, flip angle = 8°, inversion time (TI) = 764 ms, field of view (FOV) = 256 mm × 256 mm, matrix size = 256 × 256, 160 contiguous slices, slice thickness = 1 mm; number of excitations (NEX) = 1] images, axial T_2_-weighted FLAIR (TR = 11,000 ms, TE = 140 ms, TI = 2800 ms, flip angle = 90°, FOV = 250 mm × 250 mm, matrix size = 280 × 202, 40 contiguous slices, slice thickness = 3 mm, interslice gap = 0.6 mm, NEX = 1) images, axial T${}_{2}^{*}$-weighted gradient-echo [TR = 696 ms, TE = 23 ms, flip angle = 18°, FOV = 250 mm × 200 mm, matrix size = 252 × 160, 22 slices, slice thickness = 5 mm, interslice gap = 1 mm, NEX = 2] images, and diffusion-weighted imaging (DWI) volumes using an axial single-shot echo planar imaging sequence [TR = 9,394 ms, TE = 89 ms, FOV = 256 mm, matrix size = 128 × 128, 50 slices, slice thickness = 3 mm, no gap, NEX = 3, diffusion sensitizing gradients applied along 15 non-collinear directions using b value of 0 (b_0_ image) and 1,000 s/mm^2^]. An experienced neuroradiologist visually checked all images for possible artifacts prior to image processing.

### 2.3. MRI feature extraction

**Figure 1:**
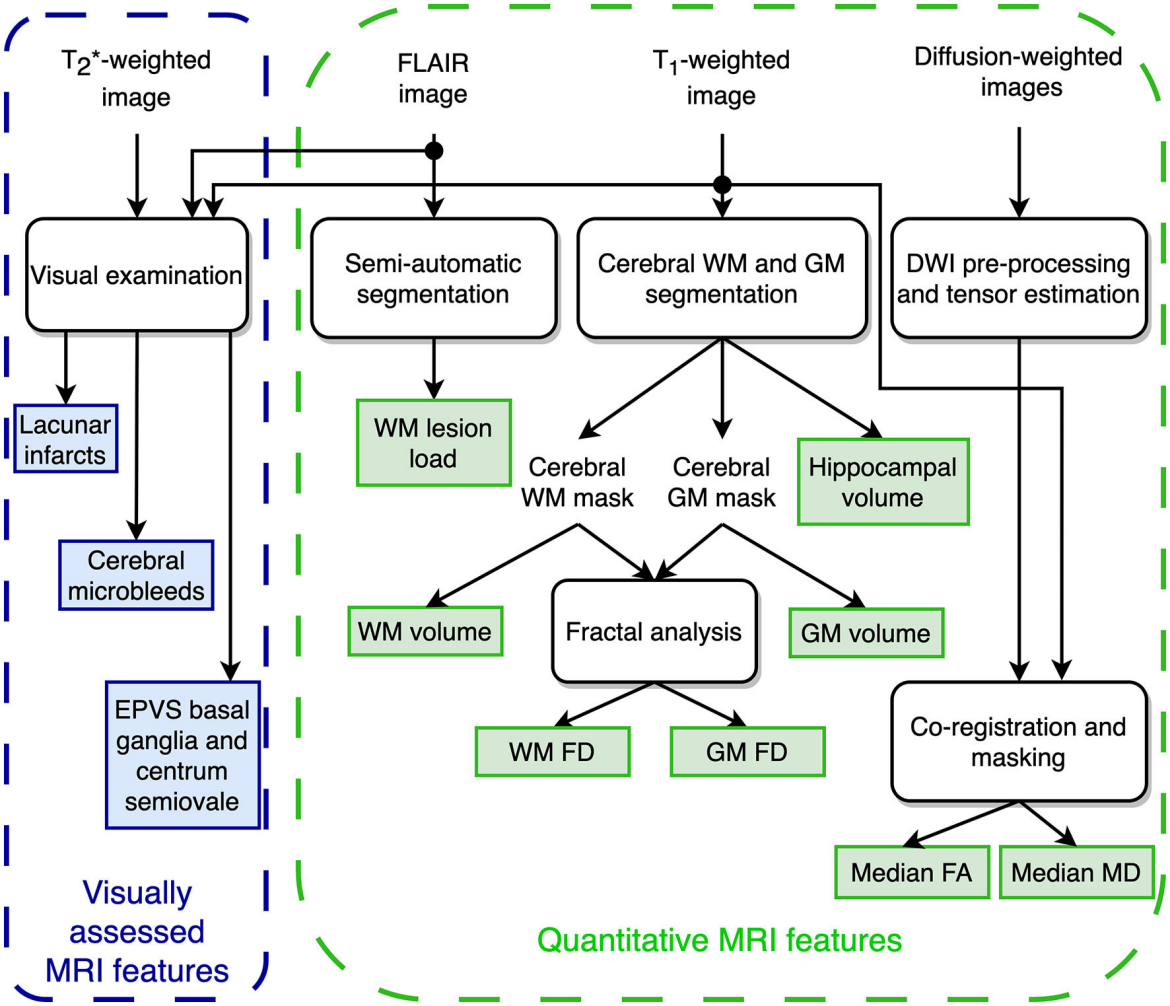
Schematic representation of the MRI features extraction for predicting the transition to dementia.

Figure 1 shows the extraction procedure for the brain MRI features considered in the present investigation and partially described in detail in a previous study (Pantoni et al., 2019).

#### 2.3.1. Visually assessed MRI features

An experienced observer visually assessed the number of lacunes, cerebral microbleeds, and enlarged perivascular spaces (EPVS). He scored lacunar infarcts, defined as cavities of 3-to-10 mm in diameter, as 0 = (absent), 1 = (1–3), and 3 = (>3) and used the Microbleed Anatomical Rating Scale (MARS) (Gregoire et al., 2009) to assess the total number of microbleeds, defined as small, rounded, or circular, well-defined T_2_ hypointense focal brain lesions ranging from 2 to 10 mm in diameter. The inter- and intra-observer agreement for rating lacunar infarcts and microbleeds in patients of the VMCI Tuscany cohort was “substantial” or “almost perfect” (Valenti et al., 2016; Mascalchi et al., 2019).

Finally, he assessed the EPVS in the basal ganglia and the centrum semiovale. These were defined as small, sharply delineated structures with cerebrospinal fluid intensity; they followed the orientation of the perforating vessels, ran perpendicular to the brain surface, and were < 3 mm wide. EPVS were scored as 0 = (absent), 1 = ( ≤ 10), 2 = (11–20), 3 = (21–40), and 4 = (≥40).

#### 2.3.2. Quantitative MRI features

The WM T_2_ hyperintensities were quantitatively assessed by computing the lesion load using a semiautomatic segmentation technique based on user-supervised local thresholding (Jim 5.0, Xinapse System, Leicester, UK; www.xinapse.com/Manual/). A single operator outlined the T_2_ hyperintense WM lesions on FLAIR images. The WM lesion load was then calculated by normalizing the total volume of lesions by the individual's cerebral WM volume.

##### 2.3.2.1. Volumes of the cerebral WM, cortical GM, and hippocampus

The FreeSurfer image analysis suite v. 5.3 (http://surfer.nmr.mgh.harvard.edu/) performed cortical reconstruction and volumetric segmentation of the WM, cortical GM, and hippocampus on T_1_-weighted images (Fischl, 2012). We applied the correction procedures for segmentation and surface reconstruction errors, as proposed by the FreeSurfer developers, to FreeSurfer outputs. They involve editing the brain and WM masks, adding control points, and re-running the FreeSurfer pipeline (https://surfer.nmr.mgh.harvard.edu/fswiki/FsTutorial/TroubleshootingData). To correct all defects, manual editing and re-running were performed by the same operator up to three times (McCarthy et al., 2015). Given the inherently hemispheric nature of the hippocampus, we computed the volumes of the hippocampus, WM, and cortical GM in the left and right hemispheres separately. Then, we calculated the average value of the volume of each structure in the left and right hemispheres and normalized these averages to the estimated intracranial volume (eTIV).

##### 2.3.2.2. Microstructural changes of the cerebral WM

DWI volumes were subjected to head motion and eddy current distortion correction using FDT (FMRIB's Diffusion Toolbox 2.0), a component of FSL 4.1.9 (Smith et al., 2004). Subsequently, brain tissue was extracted using BET (Smith, 2002). The b-matrix was reoriented by applying the rotational part of the affine transformation employed during the eddy-correction step (Leemans and Jones, 2009). A tensor model was then fitted to the raw data using a constrained non-linear least squares procedure implemented in the CAMINO package (Cook et al., 2006). Any residual non-positive definite tensors in isolated regions, primarily located at the edge of the brain, were eliminated through tensor interpolation in the log-euclidean domain (Arsigny et al., 2006). Finally, fractional anisotropy (FA) and mean diffusivity (MD) were computed from the estimated tensor field. Median MD and FA values of the cerebral WM were computed using a previously described procedure (De Stefano et al., 2006; Mascalchi et al., 2014).

##### 2.3.2.3. WM and cortical GM fractal analysis

Among different methods to compute the FD, we selected the 3-D box-counting algorithm (Russell et al., 1980), a fairly direct and reliable method to analyze fractal objects. The algorithm involves overlaying a grid of cubes of side length *s* onto a binary segmentation of a brain structure, counting the number of cubes *N(s)* needed to enclose the structure, and repeating this process for different values of *s*. To prevent any systematic influence of the grid placement, for each *s* value, we applied 20 uniformly distributed random offsets to the grid origin, and the relative box count was averaged to obtain a single *N(s)* value (Goñi et al., 2013). The FD of the structure is then computed by modeling the data points *N(s)* vs. *s* in a log-log plane as a linear regression function and calculating the absolute value of the slope of the regression line. In the natural scale, the FD, a measure of space-filling, is the exponent (sign changed) of a power law that describes the relationship between the number of cubes enclosing the structure and their side length.

Generally, a natural object such as a brain structure shows its fractal properties in a limited interval of spatial scales, named the fractal scaling window, which is unknown a priori. Therefore, we applied an automated selection of spatial scales for each brain region, searching for the interval of spatial scales in which the linear regression shows the best fit, as measured by the rounded coefficient of determination adjusted for the number of data points (R${}_{\text{adj}}^{2}$). The fractal analysis was carried out using the fractalbrain toolkit version 1.1 (Marzi, 2023) (freely available at https://github.com/chiaramarzi/fractalbrain-toolkit) and described in detail in Marzi et al. (2020).

In this study, we examined the fractal properties of both WM and cortical GM by calculating and averaging the FD from both the left and right hemispheres of each structure.

#### 2.3.3. Machine learning system

To forecast the transition to dementia, we used an explainable machine learning (ML) framework fed by baseline demographical, neuropsychological, visual, and quantitative MRI features. During the training phase, missing values in the data were imputed by replacing them with the average value of the corresponding feature. Additionally, standardization was performed by rescaling each feature to have a mean of zero and a variance of one. These imputation and standardization techniques were exclusively learned during the training phase and subsequently applied in the validation and testing phases, leading to an unbiased generalization performance.

**Figure 2:**
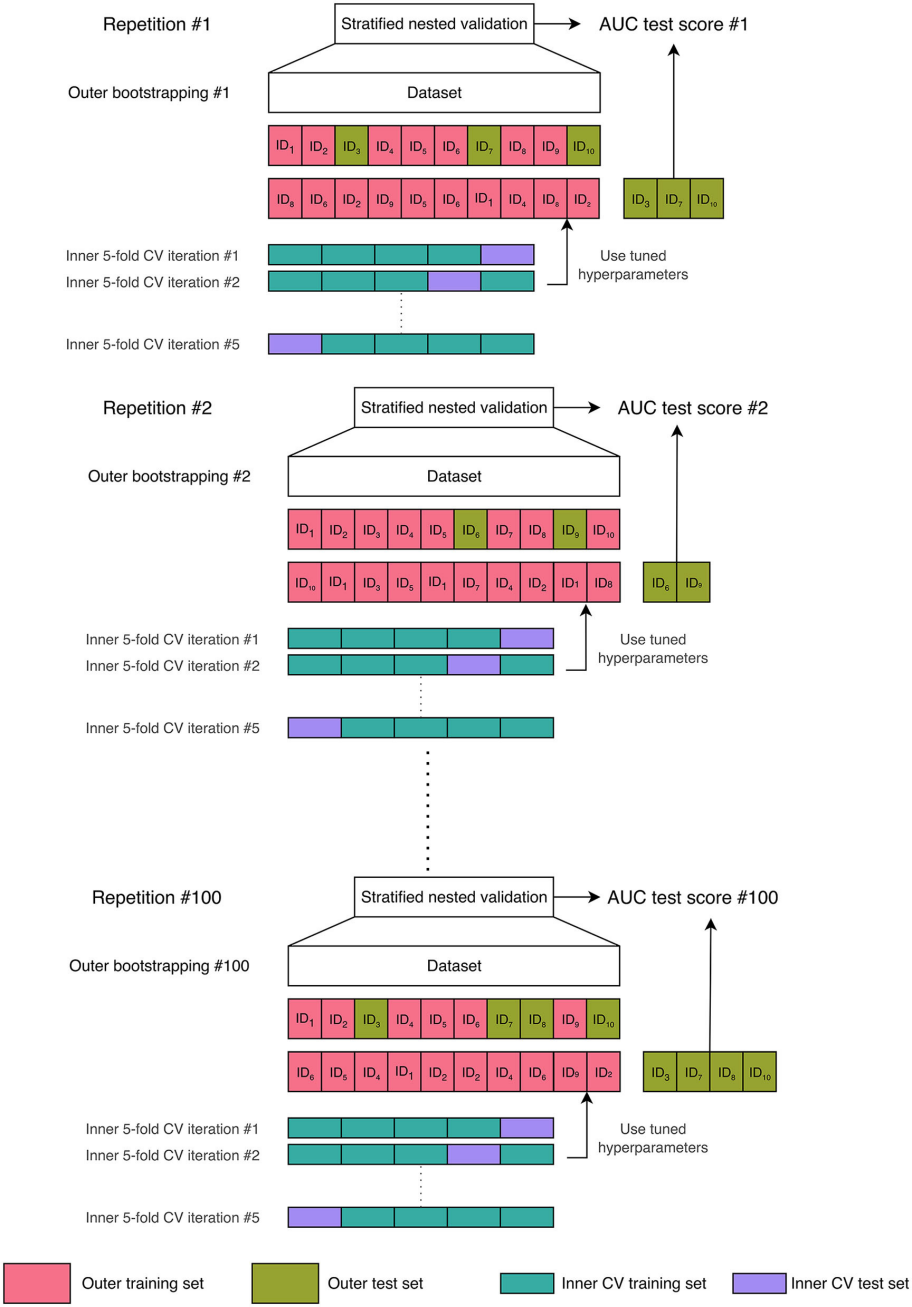
Machine learning validation scheme: 100-times repeated stratified nested validation procedure. In the figure, we chose a dataset comprising ten samples to provide a comprehensive illustration of the bootstrap resampling procedure with replacement. When applying bootstrap resampling to our actual dataset, which contains 64 subjects, we obtain an outer training set consisting of 64 instances (some of which are repeated) and an outer test set that comprises the unique instances not included in the training set, referred to as the out-of-bag samples. The outer training set was then used for an inner subject-level 5-fold cross-validation (CV) for hyperparameter optimization.

The explainable ML framework was trained, validated, and tested through a repeated stratified nested validation procedure (Figure 2). Nested validation is a technique that reduces the possibility of overfitting and model hyperparameter optimization, along with estimating the generalization error on unseen data (Müller and Guido, 2016). We chose bootstrap resampling for the outer split and a 5-fold cross-validation (CV) for the inner loop. We selected a number of folds equal to 5 because it offers a favorable bias-variance trade-off (Hastie et al., 2013). In detail, for each repetition of the bootstrap resampling, the entire dataset was divided by sampling—with replacement—the instances contained in the original dataset to form an outer training set. The outer test set included the unique instances that were not selected for the training set, i.e., the out-of-bag samples. The outer training set was then used for hyperparameter optimization through an inner subject-level 5-fold CV. The subject-level splitting ensures that the repetitions present in the outer training set are either in the inner training set or in the inner validation set, preventing data leakage (Yagis et al., 2021). Once the combination of hyperparameter values that minimized the out-of-sample prediction error (Hastie et al., 2013) had been found in the inner CV, the model with that combination of hyperparameters' values was re-trained on the outer training set and tested on the unseen outer test set, thus preventing any form of peeking effect (Diciotti et al., 2013). The stratified sampling ensured that samples possessing a particular characteristic, i.e., the transition to dementia, were selected in the same proportion in the training, validation, and test sets as they existed in the entire dataset. The stratified nested validation was repeated 100 times with different bootstrap data splitting to attenuate the dependencies of the model from the training data, along with reducing performance estimation variance while maintaining a minimal bias (Molinaro et al., 2005; Kim, 2009).

The explainable ML framework utilized in this study employed an Extreme Gradient Boosting Classifier (XGboost) model. This model, based on tree-based machine learning, has demonstrated effectiveness in addressing various recent challenges in the field of machine learning (Chen and Guestrin, 2016). The hyperparameters of the model were selected through a random search within the inner CV process. The hyperparameter space was defined as follows: the minimum loss reduction required for further partitioning a leaf node of the tree γ ∈s (0.6, 0.7, 0.8), the subsample ratio of columns used when constructing each tree *colsample_bytree* ∈ (0.25, 0.5, 0.75, 1), the maximum depth of a tree *max_depth* ∈ (2, 3, 4), the minimum number of instances required in each node *min_child_weight* ∈ (2, 3, 5), the number of decision trees *n_estimators* ∈ (5, 10, 20, 100), and the ratio of training data randomly sampled before growing trees subsample ∈ (0.1, 0.2, 0.4).

For each repetition of the stratified nested validation, the classifier's performance was evaluated on the outer test set using the area under the receiver operating characteristic (ROC) curve (AUC). The mean AUC and the 90% confidence interval (CI) were reported as the final performance. To verify whether the performance of our classifier was significantly superior to that of a random guessing classifier (Fawcett, 2006), we compared the AUC values with the value 0.5, i.e., the chance-level performance, through a one-tailed Wilcoxon signed rank with a significance level of 5%. By considering the coordinates of the ROC curve obtained from the data of the outer test set at each repetition of the stratified nested validation, we built a median ROC curve. The optimal operating point on the median ROC curve was identified as the point with the highest Youden's index, denoted as J = sensitivity + specificity – 1 (Youden, 1950).

Furthermore, we used SHAP (Lundberg, 2017), an explainable AI technique that enables the determination of feature contributions to each model output. Each SHAP value represents a real number associated with a particular feature of an individual sample (i.e., a subject). The sign of the SHAP value indicates the direction in which the feature influences the output for a specific subject. To obtain the feature contributions, SHAP values were computed for the outer test set during each repetition of the repeated nested validation and were subsequently averaged, in absolute value, across patients (Scheda and Diciotti, 2022). Therefore, we obtained 100 global SHAP values for each feature and calculated the median over the repetitions as the final global feature importance. The global contribution of the top-ranking predictive feature was compared to the second feature of the ranking through a one-tailed Wilcoxon signed rank with a significance of 0.05. In addition to assessing the individual contributions of each feature toward predicting the transition to dementia, we also averaged the SHAP values over specific feature categories (i.e., a sum of the SHAP values of all features belonging to a category divided by the total number of features in the category). These categories included demographic features (age, sex, and education), adjusted neuropsychological scores (MoCA, TMT-A, ROC-F immediate copy, SDMT, Stroop, VS), visually assessed MRI features (lacunar infarcts, cerebral microbleeds, EPVS basal ganglia, EPVS centrum semiovale), and quantitative MRI features (WM lesion load, GM FD, WM FD, hippocampal volume, GM volume, WM volume, Median MD, Median FA). By grouping the SHAP values according to these feature categories, we gained a comprehensive understanding of their combined contributions to the prediction of the transition to dementia.

## 3. Results

**Figure 3:**
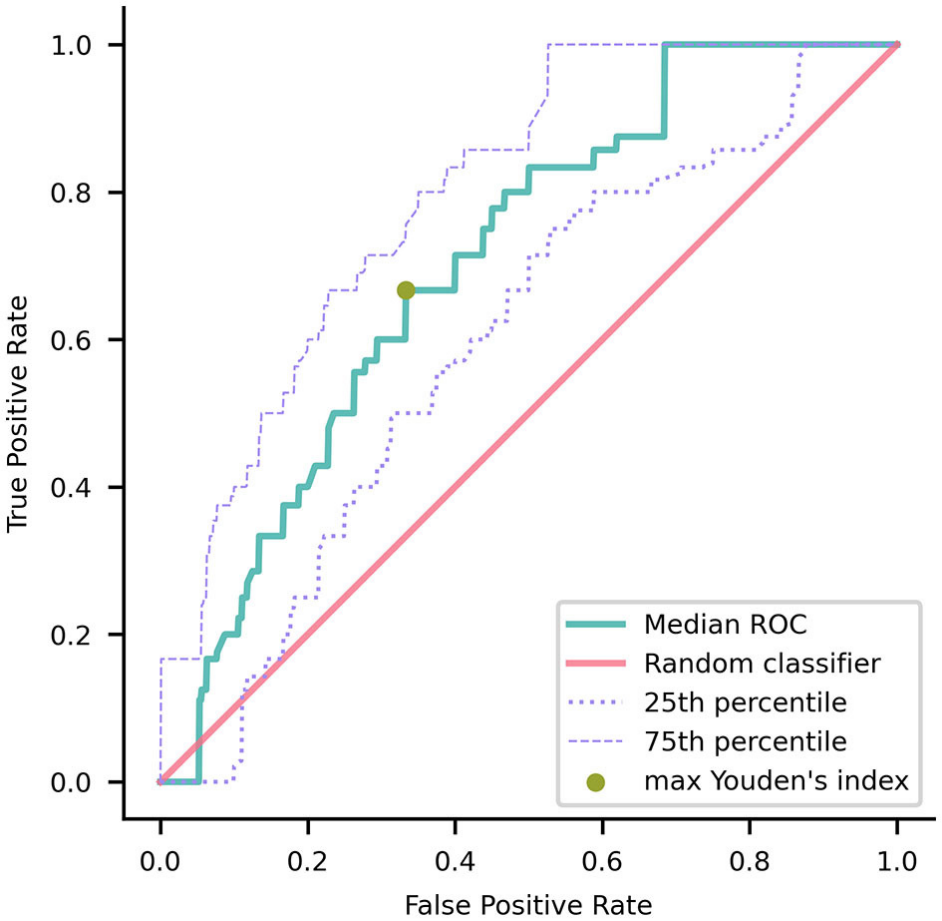
Median receiver operating characteristic (ROC) curve of the model trained using nested validation over 100 repetitions. The gold point on the ROC curve corresponds to the coordinates (0.33, 0.67) where the maximal Youden's index is achieved. The red overlay represents the ROC curve of a random classifier, serving as a reference. The dotted and dashed purple curves indicate the 25^th^ and 75^th^ percentiles, respectively.

To forecast the transition to dementia, the mean ROC AUC was 0.69 with a 90% CI of (0.53, 0.85). The AUC value of our classifier was significantly higher than the chance-level performance (one-tailed Wilcoxon signed rank *p*-value < 0.001). Through ROC curve analysis (Figure 3), we identified a specific operating point that maximized Youden's index, gaining a sensitivity of 0.67 and a specificity of 0.67.

**Figure 4:**
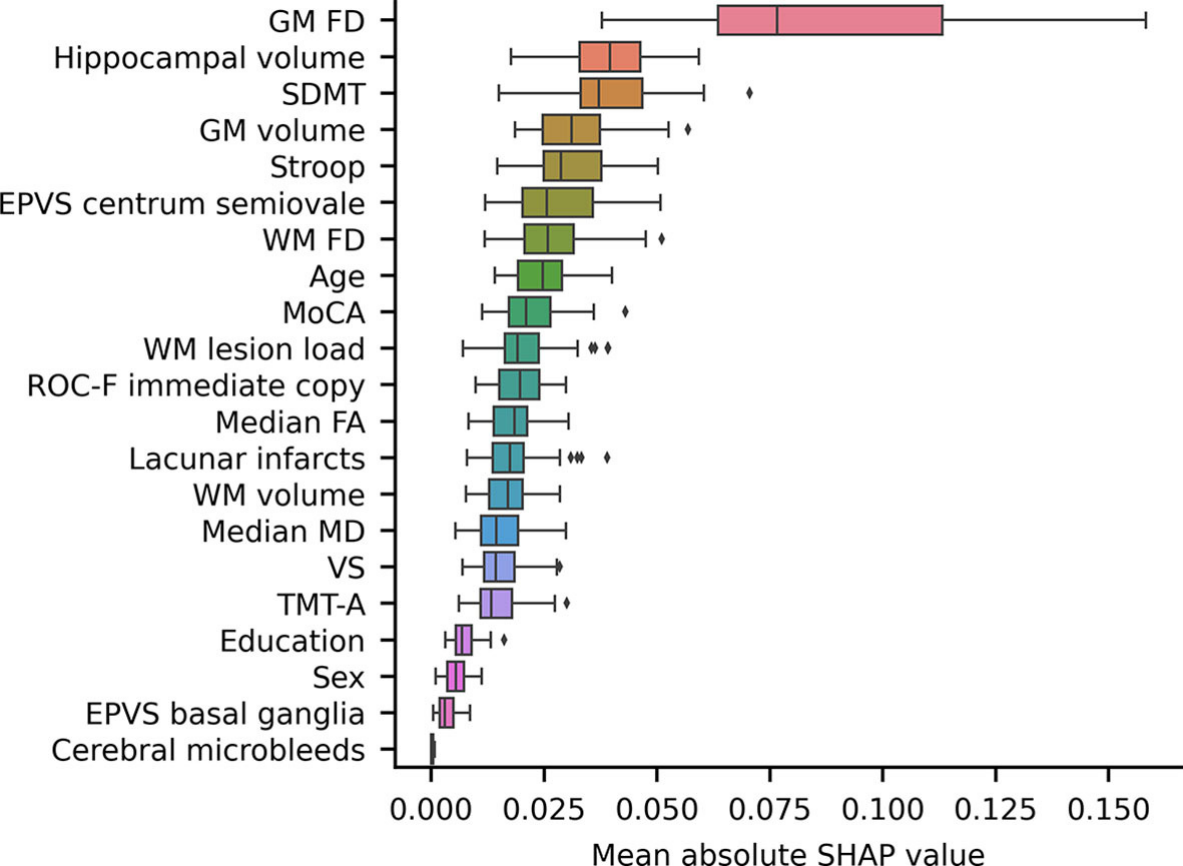
A box plot showing the mean absolute SHAP values of each feature, sorted in ascending order. The volume and the FD of a specific brain structure are defined as the average values among the left and right hemispheres. Volumes were subsequently normalized to eTIV. EPVS, enlarged perivascular spaces; FD, fractal dimension; GM, gray matter; MoCA, adjusted Montreal Cognitive Assessment score; SDMT, adjusted symbol-digit modality test score; WM, white matter.

**Figure 5:**
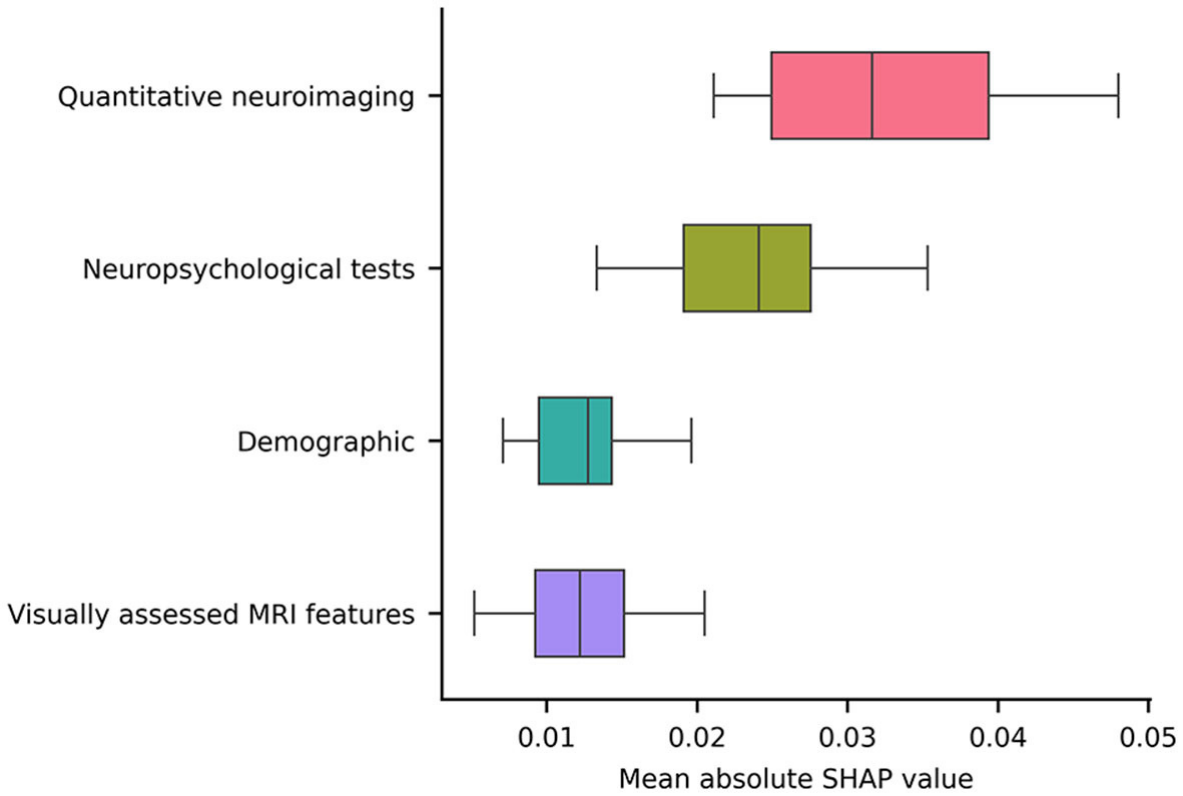
A box plot illustrating the averaged absolute SHAP values over each category (i.e., a sum of the SHAP values of all features belonging to a category divided by the total number of features in the category).

**Figure 6:**
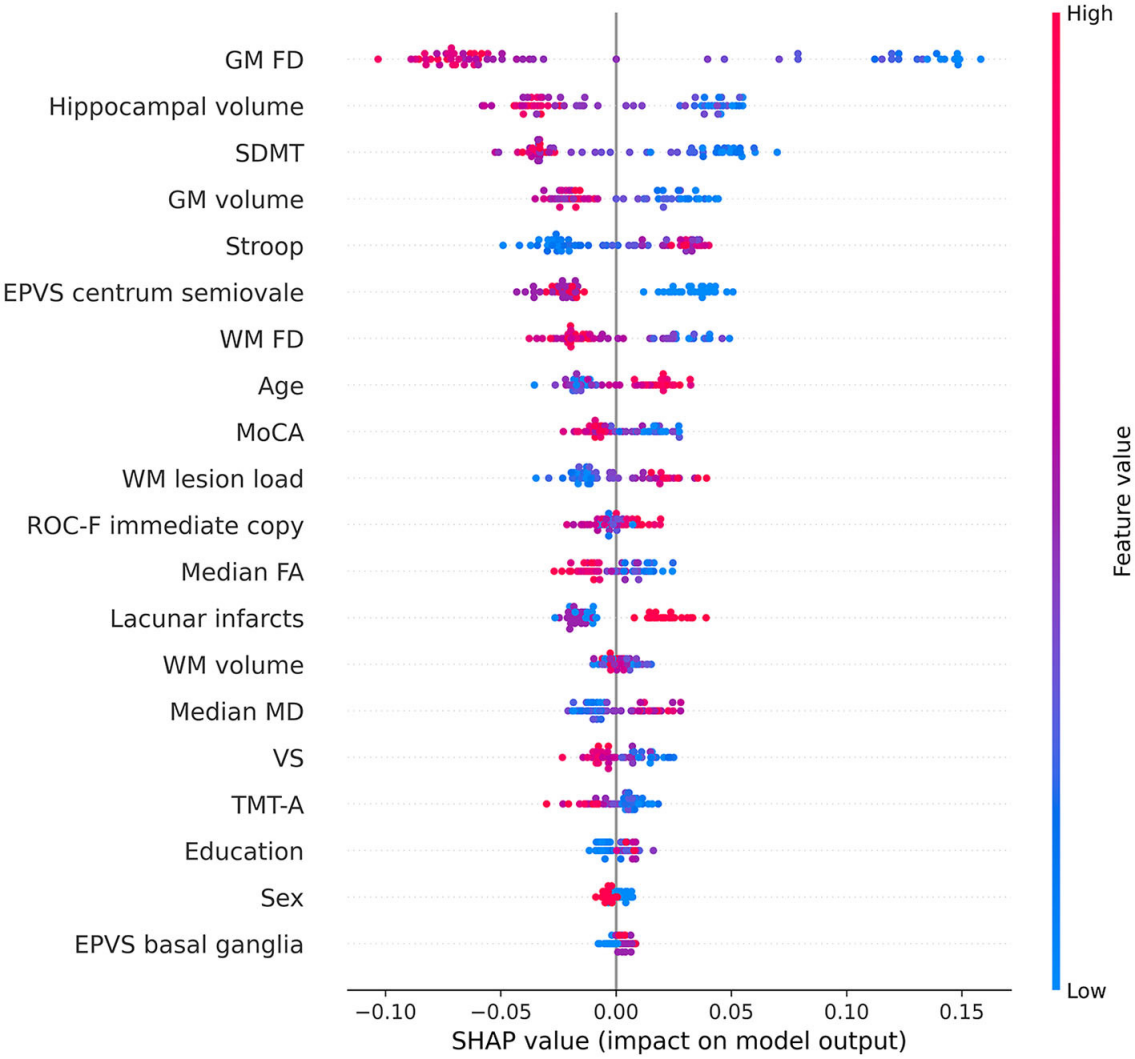
Beeswarm summary plot depicting representative SHAP values. Each feature row for each sample (i.e., subject) is represented by a single dot, with the *x* position determined by the corresponding SHAP value. Dots accumulate along each feature row to indicate density. The color of each dot represents the original value of the feature. The volume and the FD of a specific brain structure are defined as the average values among the left and right hemispheres. Volumes were subsequently normalized to eTIV. EPVS, enlarged perivascular spaces; FA, fractal anisotropy; FD, fractal dimension; GM, gray matter; MD, mean diffusivity; MoCA, adjusted Montreal Cognitive Assessment score; ROC-F, adjusted Rey-Osterrieth Complex Figure immediate copy score; SDMT, adjusted symbol-digit modality test score; TMT-A, adjusted trail making test-A score; VS, adjusted visual search score; WM, white matter.

Notably, the GM FD was the top-ranking predictive feature (Figure 4). The median absolute SHAP value of the GM FD was significantly higher than that of the second-ranking feature, i.e., hippocampal volume (one-tailed Wilcoxon signed rank *p*-value < 0.001). SDMT score, cortical GM volume, Stroop score, EPVS centrum semiovale, WM FD, age, MoCA score, and WM lesion load were the main important predictive features. The aggregated quantitative neuroimaging features exhibited superior predictive capabilities compared to visually assessed MRI features (Figure 5). Figure 6 illustrates the visualization of mean SHAP values corresponding to specific features within individual samples (subjects). This visualization aims to provide a concise representation of how the dataset's features influence the model's output. Each subject is depicted by a single dot for every feature. The SHAP value of a feature determines the horizontal position of the dot, and dots accumulate along each feature's row to depict density. Colors are utilized to indicate the original feature values. In essence, this plot enables us to observe the SHAP value for each feature in every sample. In this graphical representation, a dot's color varies (from blue to pink) according to whether the feature value is high or low. Additionally, its position on the graph shifts (from the base SHAP value to the right or left) based on its influence on the model's decision (i.e., its SHAP value). As depicted in Figure 6, lower FD values, showing decreased cortical GM structural complexity, significantly affects the model's decision, guiding it toward the transition to dementia class.

## 4. Discussion

Predicting the transition to dementia in patients with MCI is of utmost importance, as it could enable the implementation of therapies aimed at slowing or halting the progression of the disease. In a previous cross-sectional study that involved the same MCI and leukoaraiosis cohort as the current investigation, we observed that different combinations of MRI features were predictive of the cognitive status at baseline. Notably, the FD of the WM was consistently identified as the most frequently selected feature for this purpose (Pantoni et al., 2019). In this longitudinal investigation, we expanded our evaluation to include the same features, hippocampal volume, and diffusion indexes of the WM, which are well-established correlates of cognitive impairment (O'Sullivan, 2008; Mascalchi et al., 2013; Zeestraten et al., 2017). First, we wish to point out that the overall predictive performance achieved by a series of demographic, neuropsychological, and MRI features was not exceptionally high, as reflected by a mean (90% CI) ROC area of 0.69 (0.53, 0.85). We speculate that this relatively modest performance might be attributed to the broad clinical-instrumental definition of VMCI used in our study, potentially including cases with concomitant and potentially prevalent AD pathology within our sample.

Interestingly, the FD of the cortical GM emerged as the most remarkable and best predictor for this transition. Furthermore, the FD and volume of the cortical GM exhibited superior predictive performance compared to the WM lesion load, diffusion-derived indices, and FD of the cerebral WM. Notably, when features of the same type were aggregated, quantitative neuroimaging features demonstrated superior predictive capability compared to neuropsychological tests, visually assessed MRI features, and demographic factors.

Our findings provide further confirmation that cortical GM is closely associated with leukoaraiosis, as demonstrated by previous studies (Lambert et al., 2015; Ye et al., 2015; Heinen et al., 2020). Moreover, our results highlight the contribution of GM atrophy to the transition to dementia in patients with MCI and leukoaraiosis (Jokinen et al., 2012, 2020; Bilello et al., 2015; Wu et al., 2019; Fan et al., 2021). Specifically, it has been observed that cortical atrophy associated with leukoaraiosis exhibits a distinct distribution in the dorsolateral prefrontal, parietal, and posterior-superior temporal cortices, differing from the cortical changes associated with normal aging (Lambert et al., 2015; Ye et al., 2015; Heinen et al., 2020). Additionally, studies have indicated a correlation between the progression of cortical atrophy and leukoaraiosis over time (Lambert et al., 2016). Furthermore, atrophy in the hippocampal and medial temporal lobes has been identified as an underlying factor contributing to cognitive deficits in patients with leukoaraiosis (Bastos-Leite et al., 2007; Jokinen et al., 2020; Chen et al., 2021; Fan et al., 2021; Sun et al., 2022) and has been associated with their transition to dementia in these individuals (Jokinen et al., 2012, 2020). The exact nature of cortical changes in relation to leukoaraiosis and SVD remains uncertain, as some studies suggest that these changes could be secondary effects of leukoaraiosis/SVD (Bastos-Leite et al., 2007; Jokinen et al., 2020; Chen et al., 2021), while others propose the involvement of a dual pathology with accompanying AD (Jellinger, 2013; Ye et al., 2015; Wu et al., 2019). Notably, our study reveals that subtle changes in cortical GM, manifested as decreased FD, better anticipate the transition from MCI to dementia compared to overt cortical atrophy. In parallel, it is well-established that “invisible” changes in terms of subtle T_2_ signal changes (Jokinen et al., 2015) or diffusion properties (Zeestraten et al., 2017; Williams et al., 2019; Egle et al., 2022) can be observed in the normal-appearing WM of patients with leukoaraiosis, and these changes are predictive of cognitive decline. In line with these findings, our study suggests that the FD of the WM may serve as an additional marker for the subtle structural changes occurring in the WM of patients with leukoaraiosis.

The findings of this study further strengthen the evidence that FD provides supplementary information beyond what is offered by other conventional structural features (Free et al., 1996; Im et al., 2006; Sandu et al., 2008a,b, 2014a,b, 2022; King et al., 2009, 2010; Madan and Kensinger, 2016, 2018; Marzi et al., 2018, 2020, 2021, 2022; Pantoni et al., 2019; Pani et al., 2022; Nazlee and Waiter, 2023) and has potential relevant practical and diagnostic implications, particularly regarding the MRI evaluation of the cortical GM. Importantly, the FD measurement can be derived from standard, high-resolution 3D T_1_-weighted images commonly included in clinical MRI protocols. This means that FD assessment does not necessitate additional dedicated acquisitions, such as magnetization transfer imaging, which is capable of detecting subtle microstructural changes in the cortical GM in both inherited and sporadic AD (Ginestroni et al., 2009; Mascalchi et al., 2013). By contrast, nuclear medicine techniques for assessing cortical GM metabolism or amyloid deposits for the differential diagnosis of patients with leukoaraiosis have not been widely implemented (Ye et al., 2015; Altomare et al., 2023). Therefore, using FD measurements from standard MRI scans may represent a valuable and accessible tool in clinical practice for evaluating cortical GM alterations without requiring additional specialized imaging techniques.

We acknowledge several limitations in our study. First, the relatively small sample size and the fact that the study was conducted at a single center may affect the generalizability of our findings. The sample was collected in a highly qualified referral university hospital where patients fulfilling admission criteria were consecutively identified and carefully evaluated before enrollment. Of course, this cannot support the full generalizability of the results. Therefore, to enhance the robustness and generalizability of the results, further validation in independent samples would be beneficial. Second, the consideration of whole brain structures rather than regional FD differences does not allow for the demonstration of the distributed microstructural or overt changes that are known to occur in vascular MCI and dementia. Finally, longitudinal MRI data would be valuable to better elucidate the underlying mechanisms. Unfortunately, such longitudinal data are not available for our study.

In conclusion, our study highlights that the transition to dementia from MCI in patients with leukoaraiosis is associated with subtle alterations in both the cerebral cortical GM and WM, as reflected by altered FD. Notably, our findings suggest that the FD changes observed in the cortical GM exhibit a stronger predictive value for future transitions compared to other brain measurements. The FD of the cortical GM emerges as a biomarker that is potentially more sensitive than other brain measurements for predicting the transition to dementia.

## Data availability statement

The data analyzed in this study is subject to the following licenses/restrictions: The participants of this study did not give written consent for their data to be shared publicly. Requests to access these datasets should be directed to SD, stefano.diciotti@unibo.it.

## Ethics statement

The studies involving humans were approved by Ethics Committee of Careggi Hospital. The studies were conducted in accordance with the local legislation and institutional requirements. The participants provided their written informed consent to participate in this study.

## Author contributions

MM and SD are jointly supervised this work. ES performed the neuropsychological evaluation. AP and MM visually assessed MR images. AG assessed white matter hyperintensities in FLAIR images. CM and SD processed MR images and implemented the fractal analysis. RS performed the machine learning analysis. CM, MM, and SD wrote the first draft of the manuscript. All authors interpreted the results, contributed to manuscript revision, read, and approved the submitted version.
